# The Impact of Maternal Diet during Pregnancy and Lactation on the Fatty Acid Composition of Erythrocytes and Breast Milk of Chilean Women

**DOI:** 10.3390/nu10070839

**Published:** 2018-06-28

**Authors:** Cynthia Barrera, Rodrigo Valenzuela, Rodrigo Chamorro, Karla Bascuñán, Jorge Sandoval, Natalia Sabag, Francesca Valenzuela, María-Paz Valencia, Claudia Puigrredon, Alfonso Valenzuela

**Affiliations:** 1 Department of Nutrition, Faculty of Medicine, University of Chile, Av. Independencia 1027, Independencia, Casilla 70000, Santiago 8380453, Chile; cynthia.barrera@gmail.com (C.B.); rodrigochamorro@med.uchile.cl (R.C.); kbascunan@med.uchile.cl (K.B.); n.sabag.c@gmail.com (N.S.); francesca.valenzuelar@gmail.com (F.V.); mpvalencia@ug.uchile.cl (M.-P.V.); 2 Lipid Center, Institute of Nutrition and Food Technology (INTA), University of Chile, Av. El Líbano 5524, Macul, Santiago 8380453, Chile; jsandoval@hcuch.cl (J.S.); cpuigrredon@hcuch.cl (C.P.); avalenzu@inta.uchile.cl (A.V.); 3 Obstetrics and Gynecology Department, Clinical Hospital of the University of Chile, Av. Santos Dumont 999, Independencia, Santiago 8380453, Chile

**Keywords:** pregnancy, breast milk, lactation, maternal diet, *n*-6 and *n*-3 polyunsaturated fatty acid, docosahexaenoic acid

## Abstract

Maternal diet during pregnancy is relevant for fatty acid supply during fetal life and lactation. Arachidonic (AA) and docosahexaenoic (DHA) acids are also relevant for the normal growth and development of brain and visual system. AA and DHA provided by the mother to the fetus and infant are directly associated with maternal dietary intake and body stores. Our aim was to evaluate the impact of maternal diet, specially referring to the quality of fatty acid intake, in a sample of Chilean women during last stage of pregnancy and across the lactation period. Fifty healthy pregnant women (age range 20–33 years) were studied from the 6th month of pregnancy and followed until 6th month of lactation period. Diet characteristics were evaluated through food frequency questionnaires. Fatty acids composition of erythrocyte phospholipids and breast milk samples was assessed by gas-liquid chromatography. Overall, women had high saturated fatty acids intake with sufficient intake of mono- and polyunsaturated fatty acids (PUFA). Diet was high in *n*-6 PUFA and low in *n*-3 PUFA (mainly DHA), with imbalanced *n*-6/*n*-3 PUFA ratio. Erythrocytes and breast milk DHA concentration was significantly reduced during lactation compared to pregnancy, a pattern not observed for AA. We concluded that is necessary to increase the intake of *n*-3 PUFA during pregnancy and lactation by improving the quality of consumed foods with particular emphasis on its DHA content.

## 1. Introduction

Arachidonic acid (C20:4*n*-6, AA) and docosahexaenoic acid (C22:6*n*-3, DHA) are long chain polyunsaturated fatty acids (LCPUFA) that have a relevant role in different metabolic and physiological process during embryonic and fetal development, and the first years of life [1]. Highest concentrations of AA and DHA are found in nervous system, particularly in brain and retina, specifically in the phospholipids of cell membranes [2,3]. AA and DHA have an active role in brain development during neurogenesis, synaptogenesis, neuronal migration, neuronal differentiation, and also in gene expression and in the general metabolic energy status [4]. AA is formed from the precursor linoleic acid (C18:2*n*-6 LA) and DHA from the precursor alpha-linolenic acid (C18:3*n*-3, ALA) [5]. Humans can synthesize AA and DHA, mainly in the liver, through a complex metabolic process that includes different enzymatically catalyzed desaturations and elongations of their respective metabolic precursors (LA and ALA); Δ-5 and Δ-6 desaturases are the most relevant enzymes participating in these processes [6].

Hepatic synthesis of AA and DHA is a fundamental metabolic process necessary to ensure the constant supply of these LCPUFA to other tissues [7]. Synthesis of AA and DHA in women is more efficient than in men due the active positive control of estrogens over desaturase activities [8]. Women, in addition, can store LCPUFA during pregnancy and lactation to ensure an adequate flow of AA and DHA to the fetus and newborn [9]. During pregnancy, the transport of AA and DHA from the mother to embryo and fetus is facilitated by specific transporter proteins that enhance the transfer of these fatty acids through the placenta [10]. During this period, women can also incorporate AA and DHA in their diet by eating eggs and meat (as sources of preformed AA) and fatty fish (i.e., tuna, mackerel, and salmon, among others) as a sources of preformed DHA [11]). However, in western countries DHA intake from fish consumption is very low compared to the intake of LA (from vegetable oils) and AA (from eggs and meat) [11]. In addition to the high dietary intake of *n*-6 fatty acids, the synthesis of AA from LA when this fatty acid is highly consumed is more efficient than the synthesis of DHA from ALA [12].

Several studies have established the relevance of the fatty acid composition of breast milk and its direct association with the diet of the mother during pregnancy and lactation [13,14,15]. The aim of our research was to evaluate the impact of maternal diet, with specific reference to the quality of fatty acids intake, in a sample of Chilean women during the last stage of pregnancy and across the lactation period. The fatty acid composition of erythrocyte phospholipids (during pregnancy and lactation) and of breast milk (during the first sixth months of lactation) were assessed as analytical criteria for *n*-6 and *n*-3 LCPUFA availability.

## 2. Materials and Methods

### 2.1. Study Design and Subjects

Pregnant women (*n* = 50) who attended the Obstetrical and Gynecology Health Service of the Clinical Hospital, University of Chile, Santiago, Chile were included in the study. Inclusion criteria were women between 20 and 33 years; gestational age at least 22 and up to 25 weeks according to the date of the last menstrual period and confirmed by ultrasound; and history of successful lactation. Exclusion criteria were women with history of drug or alcohol consumption; with current consumption of *n*-6 and/or *n*-3 LCPUFA supplements; who were underweight (as defined by the Chilean chart for pregnant women [16]); who had a history of twins; who had been diagnosed with chronic diseases such as diabetes, arterial hypertension, or other illnesses that could affect fetal growth. Recruited women mainly belonged to the low and middle socioeconomic status according to the European Society for Opinion and Marketing Research (ESOMAR) [17]. All women were of Hispanic origin. 

At the time of recruitment, all women who fulfilled the inclusion criteria were given general information about the study, and a dietitian explained the objectives and main characteristics of the research. The protocol was reviewed and approved by the Institutional Review Board of the Faculty of Medicine, University of Chile (Protocol #073-2011), and by the Ethics Committee of the Clinical Hospital, University of Chile (Protocol #507/11). All information regarding the study was given to each participant who voluntarily agreed to participate and signed the informed consent.

### 2.2. Clinical and Nutritional Assessment

Participants were subject to a clinical evaluation when incorporated into the study. A physician and a nurse assessed each woman regarding her health by following the standard clinical approach for pregnant women. Anthropometric data of weight (kg) and height (m) were assessed to determine body-mass index (BMI, kg/m^2^), which was then used to establish the maternal nutritional status according to gestational week following the Chilean reference [17]. Energy and nutrient requirements were established according to WHO criteria [18] and recommended dietary intakes according to the American Institute of Medicine, 2001 [19].

### 2.3. Dietary Intake

All women were interviewed by trained dietitians at the enrollment of the study and asked to report all groups of foods consumed at the first week after delivery and during the six-month of lactation, using a food frequency questionnaire. In addition to this questionnaire and in order to improve the estimation of eaten foods, dietitians used a photographic atlas of commonly consumed foods in Chile [20], which is a validated graphic instrument that helps to estimate the amount of each food/beverage consumed. Food intake data were checked by contrasting the energy/nutrient intake data composition with dietary questionnaires, identifying potential under- or over-reports. In that case, a careful review of each food frequency questionnaire was done. Dietary data were grouped into nine food groups (cereals, fruits and vegetables, dairy, meats and eggs, legumes, fish and shellfish, high-fat foods, oils and fats, sugars and processed foods), according to dietary analysis previously reported by Bascuñán et al. [21]. Dietary data was analyzed using the software Food Processor SQL^®^ (ESHA Research, Salem, OR, USA), to calculate the daily intake of energy and nutrients. Nutritional composition of foods was obtained using a database from the USDA National Nutrient Database for Standard Reference, which also incorporated information from locally generated nutrient composition data.

### 2.4. Collection and Fatty Acid Analysis from Erythrocytes and Breast Milk Samples

Blood samples were obtained at the enrollment, immediately after the delivery and at the 1st and the 6th month after lactation. The samples were immediately centrifuged to obtain the erythrocyte fraction (3000× *g* for 10 min at 20 °C) and then frozen at −80 °C until further analysis. Breast milk was extracted by the mothers themselves after the infant had been fed for at least 2 min and was collected in plastic vials. Breast milk samples (5 mL) were collected monthly from the 1st until the 6th month of lactation. Once collected, the samples were immediately frozen at −80 °C until further analysis. Details of the analysis of fatty acids of erythrocyte phospholipids and breast milk samples were previously described by Valenzuela et al. [15].

### 2.5. Statistical Analyses

Dietary data were checked by contrasting the energy/nutrient intake data composition with dietary questionnaires, identifying potential outliers. In that case, a careful review of each food frequency questionnaire was done. After a descriptive analysis, the distribution of variables was evaluated using the Shapiro–Wilk test. Results are expressed as the mean ± SD. Assessment of significant differences between mean values was performed by one-way ANOVA and with Bonferroni post-hoc test. Statistical significance was set at an alpha level of 5%. For all analyses, the statistical software used was SPSS v.24.0 (Chicago, IL, USA). 

## 3. Results

### 3.1. Background and Anthropometric Characteristics of the Sample

Table 1 shows the main background characteristics of the sample (age, socio-economic status (SES), weight, BMI, nutritional status, and gestational age). 68.2% of women belonged to the SES medium. Regarding the nutritional status, 37.8% were overweight and 11.1% obese; therefore, 48.9% of women exhibited overnutrition at the study enrollment. 

Table 2 shows the anthropometric characteristics of women during the pregnancy and lactation period. Significant modification was observed in the weight and BMI during the time course of the study. The weigh and BMI were higher (*p* < 0.05) than delivery compared with other times evaluated. At the beginning of the study, 4.4% were underweight. At the end of the study, 46.7% of women were overweight and 16.1% obese; therefore, 62.8% exhibited overnutrition, and no underweight was observed. The 45% of deliveries were after cesarean, condition that may modify the milk production in amount and nutritional quality (not evaluated in the present study). At 6th month of lactation, infant presented a normal increment of weight and height. An important aspect is the increase in weight of the women from the 6th month of pregnancy until the delivery (12.2 kg on average), along with the weight of infant at birth, which on average was 4.25 kg.

### 3.2. Daily Intake According to Food Groups during Pregnancy and Lactation

Table 3 shows the dietary intake during the period studied. As expected at 6th month of lactation, a general reduction of food intake compared to the evaluation at 6th month of pregnancy was observed not produced. However, fruits and vegetables, fish and seafood, oil and fats, and sugar and processed foods consumption were reduced (*p* < 0.05) at the 1st and 6th month of lactation when compared to the 6th month of pregnancy. In addition, consumption of dairy, meat, and eggs was reduced (*p* < 0.05) at 6th month of lactation. Consumption of high-fat foods was not significantly modified during the study. A reduction (*p* < 0.05) of the ingestion of fish and seafood (39.3% at the 1st month of lactation and 54% at the 6th month of lactation) compared to the 6th month of pregnancy was observed.

### 3.3. Energy, Nutrients, and Most Relevant Fatty Acid Intake during Pregnancy and Lactation

Table 4 shows the energy, nutrients, and the most relevant fatty acids intake of the sample. At the 1st and 6th month of lactation, a significant reduction in the energy and carbohydrate intake compared to the 6th month of pregnancy was observed. No significant modification in the intake of protein, fiber, and fat was observed. Table 4 also shows the daily intake of the most relevant fatty acids during pregnancy and lactation. During the study, women had a high intake of saturated fatty acid (SFA), total *n*-6 fatty acids, LA, and AA, together with an adequate intake of total monounsaturated fatty acid (MUFA) and total polyunsaturated fatty acid (PUFA) and a low intake of total *n*-3 fatty acids ALA, EPA, and DHA. At the 1st month of lactation a significant reduction of the intake of EPA and DHA (50% and 33%, respectively) was produced, and at the 6th month this reduction was 50% for both LCPUFA, compared to the values at the 6th month of pregnancy. The rest of the most relevant fatty acids and the *n*-6/*n*-3 PUFA ratio were not significantly modified.

### 3.4. Fatty Acid Composition of Erythrocyte Phospholipids

Table 5 shows the fatty acid composition of erythrocyte phospholipids at the 6th month of pregnancy, at the delivery and at the 1st and 6th month of lactation. No significant changes were produced for SFA, MUFA, PUFA, LCPUFA, and *n*-6 LCPUFA when compared to the values for the 6th month of pregnancy and for the 6th month of lactation. However, docosapentaenoic acid (C22:5, *n*-6 DPA) was significantly increased (34.2%) and DHA and total *n*-3 LCPUFA were significantly reduced for the same period (27.6% reduction for DHA and 21.6% reduction for total *n*-3 LCPUFA, compared to the 6th month of pregnancy). 

### 3.5. Composition of the Most Relevant Fatty Acids of Breast Milk during the First Six Months of Lactation

Data from Table 6 shows that the composition of the majority of the fatty acids from milk is maintained during the period studied, with the exception of DHA, LCPUFA, and *n*-3 LCPUFA, which were significantly reduced from the 4th month of lactation. It is interesting to mention that at the 6th month of lactation the levels of DHA, LCPUFA, and *n*-3 LCPUFA were even lower (*p* < 0.05) than the values observed at the 4th month of lactation. In addition, the DHA was reduced by 38.5% at the 4th month of lactation and by 64.1% at the 6th month of lactation, compared to the values obtained at the 1st month of lactation. The reduction of *n*-3 LCPUFA is reflected in the significant increase of the *n*-6/*n*-3 LCPUFA ratio. 

## 4. Discussion 

A woman’s diet during pregnancy and lactation has a fundamental role in the adequate contribution of macro and micronutrients for her infant during the fetal life and during lactation [22,23]. The tissue levels of fatty acids in a woman during pregnancy and lactation are directly related to her diet, her reserve capacity, and her metabolic utilization of fatty acids (synthesis, oxidation, transport, etc.) [24,25]. Therefore, the diet and the metabolism of fatty acids of women during pregnancy and lactation have a relevant role in determining the levels of LCPUFA present in erythrocytes and breast milk [26,27]. The availability of LCPUFA for the infant is directly related to the transfer of these fatty acids from the mother to her offspring, first through the placenta (intra-uterine life) [28] and then through lactation [29]. Regarding AA and DHA, their availability will depend on the intake of foods that provided these fatty acids [30] and/or from the capacity of the mother to form these fatty acids from their metabolic precursors [31]. According to our results we concluded that the Chilean women evaluated have a high intake of *n*-6 PUFA, LA, and AA (Table 4), and a low intake of *n*-3 PUFA, ALA, EPA, and DHA (Table 4). In this context, is also remarkable that among LCPUFA, DHA is one the fatty acid with the most important metabolic characteristics for the physiological period studied [28,32]. The low intake of foods that are natural sources of DHA, such as fish or seafood, as was observed in our study (Table 3), added to an excessive intake of foods that are high in *n*-6 fatty acids, especially LA (e.g., consumption of soy or sunflower oil), which can produce a reduction of the capacity of the mother for transferring DHA to her offspring during pregnancy and breast feeding [13,26,27]. In our sample, DHA levels in breast milk were reduced by 38.5% at the 4th month, by 48.7% at the 5th month, and by 64.1% at the 6th month of lactation compared with the 1st month of lactation (Table 6). This situation was not produced for AA and LA in erythrocytes and breast milk because of the adequate consumption of foods considered good sources of these fatty acids (Table 3). 

The reserve capacity of women for LCPUFA, particularly DHA, is sensitive to the number of pregnancies, because it is produced by a significant decrease in the tissue levels and availability of DHA after frequent pregnancies [28,30,33]. In addition, another interesting aspect to consider is the activity of the Δ-5 and Δ-6 desaturases, key enzymes for the synthesis of LCPUFA from their specific precursors [8,31]. The presence of polymorphisms in the genes encoding these enzymes may produce a lower synthesis of both *n*-6 and *n*-3 LCPUFA, but being the diet sufficient in AA, the effect of polymorphism should be more deleterious for the availability of DHA than AA [34]. Is important to emphasize that the dietary imbalance of *n*-6 to *n*-3 PUFA can lower the synthesis of *n*-3 LCPUFA, particularly DHA, because of the competition generated between the respective precursors for the active sites of desaturase enzymes [6,8,35]. An excess of *n*-6 fatty acid (such as LA) may decrease the synthesis of DHA from ALA [36]. The increment in the values for DPAn-6 (Table 5) may be a metabolic compensatory mechanism to form LCPUFA because of the reduction of the *n*-3 LCPUFA (DHA), an aspect that remains to be studied. The synthesis of LCPUFA, as well as the availability of precursors and the activity of Δ-5 and Δ-6 desaturase enzymes, is also dependent on the availability of specific nutrients, such as zinc, magnesium, calcium, vitamin B6, and vitamin C [37]. It is also interesting that while the synthesis of DHA from ALA may be sufficient for the adult human in normal physiological conditions [38], some diseases, such as non-alcoholic fatty liver, a pathology very prevalent in populations that are overweight or obese [39] (in our study, women who were overweight or obese reached 48.9% at 6th month of pregnancy), which reduces the activity of Δ-5 and Δ-6 desaturase enzymes [40], could adversely affect the synthesis of DHA, decreasing the levels of *n*-3 LCPUFA in erythrocytes and breast milk, as was observed in this work (Table 5 and Table 6). Another aspect observed in this study was the increase in weight of the women from the 6th month of pregnancy until the delivery (average 12.2 kg), in addition to the high prevalence of overweight and obese women at the end of pregnancy (62.8%), along with the birth weight of children, which, on average, was 4.25 kg. In this context, previous studies suggest that Chilean women during the fertile age show a fast and growing tendency towards obesity [41], and a high prevalence of being overweight (50%) and obese (20%) is observed in pregnant Chilean women [42]. This nutritional situation has been related to (i) the increasing tendency of the Chilean newborns to have a weight at birth higher than 4.0 kg [43] and (ii) a high number of births by caesarian intervention, which are associated with overweight and obesity of pregnant women (up to 35%) [44].

In relation to the Chilean population, Bascuñán et al. [21] reported that women in the 3rd trimester of pregnancy with a low intake of DHA showed low levels of this fatty acid in erythrocyte phospholipids. In the population evaluated in this study, it was observed that DHA levels in erythrocytes were significantly decreased at the 6th month of lactation (Table 5) and the content of DHA in breast milk also decreased (*p* < 0.05), starting from the 4th month of lactation (Table 6). In this same context, Valenzuela et al. [15] reported that in Chilean pregnant women, who included in their diet chia oil (“*Salvia hispanica* L.”, 60% ALA) instead of the traditionally consumed oils (soy and sunflower oil) from the 6th month of pregnancy and until the 6th month of lactation, it a significant increase of DHA levels in breast milk was produced, but only until the 3rd month of lactation, without modification of the AA levels during the 6th month of lactation. The same authors reported that AA and DHA in erythrocyte phospholipids were not modified during the dietary intervention [15], suggesting that the ingestion of oils with high content of ALA (such as chia oil) would not be entirely efficient to increase the levels of DHA in breast milk during a lactation period up to three months [15].

Concerning the actual background of *n*-3 LCPUFA, dietary strategies have been developed to improve the quality of the diet of women during pregnancy and lactation through educational programs focused on promoting consumption of foods that provide DHA (especially from marine origin) [45,46,47]. However, it is not easy to modify the dietary habits of women during these periods [45,47], which adds to the concern that currently exists about the contamination of foods from marine origin (heavy metals, dioxins, PGB, etc.) and the questioning in the population about “possible adverse effects” of seafood consumption [48,49,50]. It is remarkable that, in the present study, it was observed that women significantly decreased fish intake after the delivery (Table 3), even though they were advised by professional nutritionist that they should increase the consumption of fish and other foods from marine origin. In this regard, the decrease of DHA levels (*p* < 0.05) observed in erythrocytes and breast milk in women’s sample (Table 5 and Table 6) may be a concern, as it has been previously reported that higher levels of DHA in breast milk are associated with better academic performance, particularly in mathematics, in long-term studies on children [51]. Addressing this dietary and nutritional problem, various studies have used supplements containing DHA or DHA-added foods, evidencing that an increase in the intake of DHA increases the content of the fatty acid in erythrocytes and subsequently in breast milk in pregnant and lactating women [52,53,54,55]. In consistent with this same direction, the Chilean Ministry of Health has developed a food program that benefits (free of cost) all women during pregnancy and lactation, providing a dairy drink containing DHA (60 mg/200 mL of product), and recommending the intake of two daily portions (120 mg DHA/day) [56]. Although the program began in 2009, there are no current results regarding the impact of this dairy product, either on DHA levels in erythrocytes and breast milk or on scholarly performance. The results of this program are currently being assessed.

## 5. Conclusions

This study shows that Chilean women during pregnancy and lactation have a low intake of foods that are natural sources of *n*-3 fatty acids (vegetable oils, fish, and seafood) and a high intake of *n*-6 fatty acid (LA and AA). This dietary situation produced a significant reduction of DHA levels in erythrocytes and breast milk. Therefore, it is necessary to promote the consumption of foods that naturally contain DHA or are fortified with *n*-3 LCPUFA. Another alternative is direct supplementation with DHA during pregnancy and lactation through products now widely available (fish oil or krill oil capsules or microcapsules). 

## Figures and Tables

**Table 1:** Background characteristics of the women at the study enrollment.

Variable		(*n* = 50)
Age (Years)		29.4 ± 6.2
SES	High (%)	20.4
	Medium (%)	68.2
	Low (%)	11.4
Preconception Weight (kg)		64.9 ± 9.3
Preconception BMI (kg/m^2^)		24.9 ± 3.3
Nutritional Status	Underweight (%)	4.4
	Normal Weight (%)	46.7
	Overweight (%)	37.8
	Obese (%)	11.1
Gestational Age (Weeks) *		24.2 ± 3.8

Value are shown as mean ± S.D., or as a percentage (%); SES, socioeconomic status; BMI, body mass index = kg/m^2^. (*) Data taken at study enrollment.

**Table 2:** Anthropometric characteristics of the women during the pregnancy and lactation period.

Variable	6th Month of Pregnancy	Delivery	1th Month of Lactation	6th Month of Lactation
Weight (kg)	70.3 ± 9.0 ^b^	82.5 ± 10.8 ^a,c,d^	69.1 ± 9.7 ^b^	66.2 ± 9.4 ^b^
Height (m)	1.61 ± 0.1	1.61 ± 0.1	1.61 ± 0.1	1.61 ± 0.1
BMI (kg/m^2^)	27.1 ± 3.2 ^b^	31.9 ± 3.9 ^a,c,d^	26.5 ± 3.5 ^b^	25.5 ± 3.4 ^b^
Nutritional Status				
Underweight (%)	4.4	0	0	0
Normal Weight (%)	46.7	42.2	40.8	37.2
Overweight (%)	37.8	38.3	44.4	46.7
Obese (%)	11.1	19.5	14.8	16.1
Gestational age at birth (weeks)	---------	39 ± 1	---------	---------
Vaginal delivery (%)	---------	55	---------	---------
Cesarean delivery (%)	---------	45	---------	---------
Gender: male (%)	---------	53	---------	---------
Gender: female (%)	---------	47	---------	---------
Infant Weight (g)	---------	4251 ± 489 ^d^	4619 ± 619	7916 ± 852 ^b^
Infant Height (cm)	---------	47.9 ± 4.2 ^d^	53.9 ± 5.8	66.9 ± 6.4 ^b^

Values are shown as mean ± S.D., or as a percentage (%); BMI, body mass index = kg/m^2^. Statistical significance (*p* < 0.05). ^a^: Significantly different from the 6th month of pregnancy; ^b^: significantly different at birth; ^c^: significantly different from 1st month of lactation; and ^d^: significantly different from the 6th month of lactation. One-way ANOVA and Bonferroni test.

**Table 3:** Daily intake according to the food groups consumed by the women during the pregnancy and lactation period.

	Food Groups Intake (g/Day)		
Food Groups	6th Month of Pregnancy	1st Month of Lactation	6th Month of Lactation
Cereals	347.0 ± 59.8	302.7 ± 40.3	299.7 ± 31.7
Fruits and Vegetables	638.9 ± 51.2 ^b,c^	371.5 ± 54.6 ^a^	303.6 ± 38.9 ^a^
Dairy	461.4 ± 40.2 ^c^	383.6 ± 38.6	332.2 ± 27.6 ^a^
Meats and Eggs	113. 5 ± 12.5 ^c^	112.0 ± 10.6 ^c^	85.2 ± 8.4 ^a,b^
Fish and Seafood	28.5 ± 6.9 ^b,c^	17.3 ± 3.3 ^a^	13.1 ± 2.4 ^a^
Legumes	20.0 ± 4.4 ^b^	10.5 ± 2.6 ^a^	15.9 ± 4.4
High-Lipid Foods	47.1 ± 10.9	38.6 ± 9.7	37.1 ± 8.9
Oils and Fats	39.8 ± 7.3 ^b,c^	23.5 ± 5.5 ^a^	22.6 ± 4.4 ^a^
Sugar and Processed Foods	537.7 ± 53.5 ^b,c^	368.6 ± 48.6 ^a^	390.3 ± 41.7 ^a^

Value are shown as mean ± S.D. Statistical significance (*p* < 0.05). ^a^: Significantly different from the 6th month of pregnancy; ^b^: significantly different from the 1st month of lactation; and ^c^: significantly different the 6th month of lactation. The food was organized in nine groups according to the methodology described in the text. One-way ANOVA and Bonferroni test.

**Table 4:** Energy, nutrients, and most relevant fatty acid consumed by the women during the pregnancy and lactation period.

	Time of Study					
Energy/Nutrients/Fatty Acid	6th Month of Pregnancy	Adequacy (%) ^Φ^	1st Month of Lactation	Adequacy (%) ^Φ^	6th Month of Lactation	Adequacy (%) ^Φ^
Energy (kcal)	2721.4 ± 254.5 ^b,c^	126.9 ± 18.6 ^b,c^	2157.6 ± 219.3 ^a^	92.4 ± 8.9 ^a^	2110.2 ± 231.4 ^a^	96.2 ± 9.2 ^a^
Protein (g)	97.1 ± 26.6	139.6 ± 35.4	86.8 ± 29.7	121.1 ± 27.5	81.4 ± 32.6	123.6 ± 27.9
Carbohydrate (g)	442.4 ± 75.8 ^b,c^	121.8 ± 21.5 ^b,c^	278.4 ± 61.1 ^a^	83.1 ± 9.2 ^a^	261.5 ± 55.9 ^a^	82.1 ± 8.7 ^a^
Fiber (g)	32.6 ± 12.4	116.6 ± 25.8	22.3 ± 8.1	76.8 ± 14.6	25.7 ± 11.7	79.6 ± 15.8
Fat (g)	90.9 ± 31.1	129.1 ± 23.5	82.0 ± 26.6	115.4 ± 16.8	86.9 ± 28.4	118.7 ± 19.0
SFA (g)	38.5 ± 4.8	154.5 ± 44.3	32.6 ± 3.9	135.4 ± 33.2	34.8 ± 2.8	145.1 ± 36.8
MUFA (g)	24.7 ± 3.7	102.2 ± 10.5	22.9 ± 2.9	95.8 ± 9.9	25.7 ± 2.9	101.8 ±10.1
PUFA (g)	25.6 ± 3.1	98.3 ± 9.5	24.4 ± 7.4	101.1 ± 11.4	25.3 ± 7.4	95.2 ± 10.2
*n*-3 fatty acid (g)	2.96 ± 0.92	59.4 ± 5.8	2.68 ± 0.5	61.9 ± 6.8	2.75 ± 0.5	47.1 ± 5.1
*n*-6 fatty acid (g)	22.5 ± 3.23	161.8 ± 15.4	21.4 ± 2.6	155.3 ± 18.9	22.4 ± 2.6	143.3 ± 14.4
18:2*n*-6 (LA) (g)	18.5 ± 1.27	152.2 ± 19.3	18.5 ± 2.1	168.5 ± 20.3	21.1 ± 2.2	149.1 ± 14.8
18:3*n*-3 (ALA) (g)	2.78 ± 0.90	68.7 ± 10.9	2.54 ± 0.4	71.4 ± 9.3	2.68 ± 0.6	59.4 ± 8.5
20:4*n*-6 (AA) (g)	1.57 ± 0.03	191.5 ± 23.5	1.06 ± 0.02	132.5 ± 25.4	1.12 ± 0.05	145 ± 27.5
20:5*n*-3 (EPA) (g)	0.04 ± 0.01 ^b,c^	41.4 ± 8.8 ^b.c^	0.02 ± 0.005 ^a^	23.8 ± 5.3 ^a^	0.02 ± 0.005 ^a^	25.2 ± 5.0 ^a^
22:6*n*-3 (DHA) (g)	0.06 ± 0.01 ^b,c^	30.1 ± 3.8 ^b,c^	0.04 ± 0.005 ^a^	20.8 ± 2.5 ^a^	0.03 ± 0.005 ^a^	16.9 ± 1.8 ^a^
*n*-6/*n*-3 PUFA ratio	7.60 ± 0.61	-----	7.99 ± 0.64	-----	8.15 ± 0.72	-----

Values are shown as the mean ± S.D.; ^Φ^ adequacy: (nutrient intake/nutrient daily recommendation) × 100; for this nutrient, energy or fatty acid, the proposed recommendation is “as low possible while consuming a nutritionally adequate diet”. ^a^: Significantly different from the 6th month of pregnancy; ^b^: significantly different from the 1st month of lactation; and ^c^: significantly different from the 6th month of lactation. The food was organized in nine groups according to the methodology described in the text. One-way ANOVA and Bonferroni test. Saturated fatty acids (SFA) correspond to 6:0, 8:0, 10:0, 12:0, 14:0, 16:0, 18:0, 20:0 and 22:0, and 24:0. Monounsaturated fatty acids (MUFA) correspond to 14:1*n*-5, 16:1*n*-7, and 18:1, *n*-9. Polyunsaturated fatty acids (PUFA) correspond to 18:2*n*-6, 18:3, *n*-3, 20:4*n*-6, 20:5*n*-3, 22:5*n*-3, and 22:6*n*-3.

**Table 5:** Fatty acid composition in erythrocyte phospholipids of the women during the pregnancy and lactation period.

	Time of Study			
	6th Month of Pregnancy	Delivery	1st Month of Lactation	6th Month of Lactation
Fatty Acid	Fatty Acid Composition (FAME)			
C16:0	33.5 ± 3.7	32.5 ± 3.9	30.8 ± 2.9	31.4 ± 3.2
C18:0	16.2 ± 1.2	18.9 ± 1.7	17.9 ± 1.6	18.6 ± 1.7
C18:1*n*-9	11.8 ± 1.1	12.8 ± 1.6	13.5 ± 1.8	12.7 ± 1.1
C18:2*n*-6 (LA)	12.6 ± 1.0	12.8 ± 1.1	12.5 ± 1.3	12.4 ± 0.9
C18:3*n*-3 (ALA)	1.06 ± 0.1	1.09 ± 0.2	1.12 ± 0.1	1.14 ± 0.2
C20:4*n*-6 (AA)	12.9 ± 1.2	12.5 ± 1.4	12.1 ± 1.0	11.8 ± 1.3
C20:5*n*-3 (EPA)	0.98 ± 0.1	0.95 ± 0.1	1.03 ± 0.2	0.93 ± 0.2
C22:5*n*-6 (DPAn-6)	0.73 ± 0.05 ^d^	0.76 ± 0.05	0.81 ± 0.1	0.98 ± 0.1 ^a^
C22:5*n*-6 (DPAn-3)	0.57 ± 0.04	0.62 ± 0.05	0.67 ± 0.05	0.70 ± 0.1
C22:6*n*-3 (DHA)	4.16 ± 0.6 ^d^	4.03 ± 0.4	3.96 ± 0.3	3.01 ± 0.2 ^a^
SFA	52.6 ± 3.2	54.6 ± 4.3	52.8 ± 3.2	51.8 ± 3.8
MUFA	13.5 ± 1.4	15.6 ± 1.7	16.8 ± 1.9	14.6 ± 1.6
PUFA	33.9 ± 2.8	29.8 ± 2.5	30.4 ± 3.1	33.6 ± 2.9
LCPUFA	19.7 ± 1.6	19.1 ± 1.4	18.9 ± 1.2	17.6 ± 1.0
*n*-6 LCPUFA	13.7 ± 1.5	13.4 ± 1.2	13.1 ± 1.0	12.9 ± 1.1
*n*-3 LCPUFA	6.00 ± 0.6 ^d^	5.70 ± 0.5	5.80 ± 0.04	4.70 ± 0.05 ^a^
*n*-6/*n*-3 LCPUFA ratio	2.28 ± 0.05	2.35 ± 0.04	2.26 ± 0.05	2.75 ± 0.05

Data are expressed as g fatty acid per 100 g FAME and represent the mean ± SD for *n* = 60 women. Statistical significance (*p* < 0.05). ^a^: Significantly different from the 6th month of pregnancy; ^d^: significantly different from the 6th month of lactation. One-way ANOVA and Bonferroni test. The identification of saturated and unsaturated fatty acids and their relationships are shown in Table 4.

**Table 6:** Composition of most relevant fatty acid from breast milk of the women during the lactation period studied.

	Time of Study					
FA Composition	1st Month	2rd Month	3rd Month	4th Month	5th Month	6th Month
C12:0	2.75 ± 0.2	2.64 ± 0.2	2.29 ± 0.1	2.84 ± 0.2	2.55 ± 0.2	2.34 ± 0.1
C14:0	6.92 ± 0.4	6.17 ± 0.5	6.34 ± 0.4	6.19 ± 0.5	5.98 ± 0.4	5.83 ± 0.6
C16:0	25.5 ± 2.9	24.3 ± 2.5	25.1 ± 2.8	25.7 ± 3.1	24.9± 2.8	24.6 ± 2.4
C18:0	4.15 ± 0.5	4.23 ± 0.4	4.38 ± 0.6	4.56 ± 0.4	5.01± 0.5	4.09 ± 0.5
C18:1*n*-9	33.2 ± 3.9	33.1 ± 3.7	35.2 ± 4.0	37.5 ± 4.5	37.7 ± 3.5	40.7 ± 4.6
C18:2*n*-6 (LA)	18.1 ± 2.1	18.7 ± 1.9	17.2 ± 2.0	16.8 ± 1.7	17.0 ± 1.5	16.8 ± 1.3
C18:3*n*-3 (ALA)	2.12 ± 0.3	2.29 ± 0.4	1.99 ± 0.3	1.92 ± 0.4	1.85 ± 0.2	1.79 ± 0.2
C20:4*n*-6 (AA)	0.75 ± 0.1	0.79 ± 0.1	0.74 ± 0.1	0.72 ± 0.05	0.68 ± 0.1	0.70 ± 0.05
C20:5*n*-3 (EPA)	0.13 ± 0.04	0.11 ± 0.02	0.12 ± 0.03	0.09 ± 0.03	0.11 ± 0.03	0.09 ± 0.02
C22:6*n*-3 (DHA)	0.39 ± 0.04 ^d,e,f^	0.37 ± 0.04 ^d,e,f^	0.36 ± 0.03 ^d,e,f^	0.24 ± 0.02 ^a,b,c,f^	0.19 ± 0.03 ^a,b,c^	0.14 ± 0.02 ^a,b,c,d^
SFA	40.5 ± 4.6	39.5 ± 4.3	40.6 ± 3.9	39.5 ± 3.8	38.6 ± 4.8	36.9 ± 3.8
MUFA	36.9 ± 3.2	37.2 ± 3.8	38.9 ± 4.1	40.6 ± 4.0	40.7 ± 4.5	43.1 ± 4.8
PUFA	22.6 ± 2.7	23.3 ± 2.8	20.5 ± 2.5	19.9 ± 2.0	20.7 ± 1.8	20.0 ± 1.6
LCPUFA	1.46 ± 0.1 ^d,e,f^	1.40 ± 0.1 ^d,e,f^	1.36 ± 0.1 ^e,f^	1.19 ± 0.1 ^a,b,f^	1.08 ± 0.05 ^a,b,c^	0.99 ± 0.05 ^a,b,c,d^
*n*-6 LCPUFA	0.83 ± 0.1	0.85 ± 0.1	0.81 ± 0.1	0.78 ± 0.05	0.74 ± 0.05	0.72 ± 0.05
*n*-3 LCPUFA	0.63 ± 0.05 ^d,e,f^	0.55 ± 0.05 ^d,e,f^	0.55 ± 0.04 ^d,e,f^	0.41 ± 0.03 ^a,b,c,e,f^	0.34 ± 0.02 ^a,b,c,d,f^	0.27 ± 0.02 ^a,b,c,d,e^
*n*-6/*n*-3 LCPUFA ratio	1.32 ± 0.1 ^b,c,d,e,f^	1.55 ± 0.1 ^a,d,e,f^	1.74 ± 0.1 ^a,e,f^	1.90 ± 0.2 ^a,b,f^	2.18 ± 0.3 ^a,b,c^	2.67 ± 0.3 ^a,b,c,d^

Data are expressed as g fatty acid per 100 g FAME and represent the mean ± SD for *n* = 60 women. Statistical significance (*p* < 0.05). ^a^: Significantly different from the 1st month of lactation; ^b^: significantly different from the 2rd month of lactation; ^c^: significantly different from the 3rd month of lactation; ^d^: significantly different from the 4th month of lactation; ^e^: significant difference from de 5th month of lactation; and ^f^: significant difference from 6th month of lactation. One-way ANOVA and Bonferroni test. The identification of saturated and unsaturated fatty acids and their relationships are shown in Table 4.
